# Dermatoscopic Features of Naevi During Pregnancy—A Mini Review

**DOI:** 10.3389/fmed.2021.727319

**Published:** 2021-08-09

**Authors:** Ioana Cosgarea, Marco Trevisan-Herraz, Loredana Ungureanu, Iris Zalaudek

**Affiliations:** ^1^Newcastle upon Tyne Hospitals National Health Service Trust, Newcastle Oncology and Dermatology, Newcastle upon Tyne, United Kingdom; ^2^Translation and Clinical Research Institute, Newcastle University, Newcastle upon Tyne, United Kingdom; ^3^Biosciences Institute, Newcastle University, Newcastle upon Tyne, United Kingdom; ^4^Department of Dermatology, Iuliu Haţieganu University of Medicine and Pharmacy, Cluj-Napoca, Romania; ^5^Dermatology Clinic, Maggiore Hospital, University of Trieste, Trieste, Italy

**Keywords:** pregnancy, naevi, dermatoscopy, dermatoscopy in pregnancy, naevi in pregnancy

## Abstract

Changes in melanocytic naevi and development of new naevi have been reported in pregnant women. The association between pregnancy and melanoma is a controversial topic. We conducted this review to identify the dermatoscopic changes that occur in naevi during pregnancy that could facilitate in distinguishing benign from suspicious lesions. Medline, Scopus, and Embase datasets were reviewed for clinical studies on dermatoscopic characteristics of melanoma and naevus in pregnancy. Six cohort studies with a total of 258 patients with 1,167 skin lesions that were examined fulfilled the conditions to be included in the review. None of the patients developed melanoma. Development of new naevi, when reported, was observed in less than half of the participants. The most frequent observed dermatoscopic change among the studies was the increase in the number of dots. Development of new vessels, hypo- and hyperpigmentations and changes in the pigment network were common described changes. The included studies were heterogeneous not allowing head-to-head comparisons between them. Robust and larger studies of dermatoscopic evaluation of naevi in pregnant women are needed to determine high-risk dermatoscopic characteristics.

## Introduction

Melanocytic naevi undergo changes throughout the lifetime, with the total naevus count increasing in early adulthood up to midlife and thereafter decreasing due to involution (1, 2). Changes in melanocytic naevi and development of new naevi have been reported in pregnant women (3, 4). The association between pregnancy and melanoma has been a controversial topic for a long time. Also, data about pregnancy-associated melanoma (PAM), defined as melanoma that develops during pregnancy and up to 1 year postpartum, are controversial as some suggest a worse prognosis compared to melanoma in nonpregnant patients, the former being reported with a 17% higher mortality rate (3, 5). Furthermore, in a recent Swedish population-based study, melanoma was the most common malignancy during pregnancy, followed by breast and cervical cancer (6).

The identification of dermatoscopic criteria of pregnancy-related naevi changes and melanoma features would aid in the early diagnosis of a PAM. However, the literature regarding dermatoscopic changes of naevi and melanoma during pregnancy is limited and consists of case reports and small cohort studies. The aim of the present review is to provide an overview of the literature and to help identify dermatoscopic criteria of naevi and melanoma during pregnancy that could aid in the early diagnosis of PAM.

## Materials and Methods

### Search Strategy

We reviewed the Medline, Scopus, and Embase datasets for clinical studies published between 1989 and the 11th July, 2020, on dermatoscopic characteristics of melanoma and naevus in pregnancy.

We followed the PRISMA guidelines (Preferred Reporting Items for Systematic Reviews and Meta-Analysis) (7) for methodology and reporting.

The words “melanoma,” “nevus,” “pregnancy,” and “dermatoscopy” were used to identify studies examining female patients which underwent skin examination using a dermatoscope during their pregnancy. The following combinations of MeSH (Medical Subject Heading) terms and Boolean operators were applied in our Medline search: melanoma OR melanoma, amelanotic OR nevus OR skin neoplasms AND pregnancy AND dermoscopy. The following combinations of MeSH terms and Boolean operators were used for Scopus: [(“dermoscopy” OR “dermatoscopy”) AND (“pregnancy” OR “pregnant”) AND (“naevus” OR “nevus” OR “nevi” OR “melanoma”)]. Combinations of MeSH terms and Boolean operators used in our search on Embase included the following: [(nevus) OR (naevus) OR (metastatic melanoma) OR (mucosal melanoma) OR (melanoma) OR (nonmelanoma skin cancer) OR (amelanotic melanoma) OR (cutaneous melanoma) AND (dermoscopy) OR (epiluminescence microscopy) AND pregnancy].

Reference list of included articles were manually searched for further studies.

### Study Selection

Two authors (I.C. and L.U.) conducted eligibility assessment, data extraction, quality assessment, and bias assessment independently.

Only articles published in English or German were considered for further review. We included in the present review prospective studies of any design and retrospective cohort analyses. Case reports, systematic reviews, and meta-analyses were excluded from our analysis. Article eligibility was determined based on screening of titles and abstracts. Complete articles were reviewed entirely and assess for acceptability.

### Data Extraction and Quality Assessment

Data were extracted using a predesigned form. The quality of the included studies was rated using the Newcastle-Ottawa Quality Assessment Form for Cohort Studies (8) (Supplementary Table 1) and risk of bias was assessed using the Cochrane Risk of Bias Tool (9) (Supplementary Table 2).

## Results

The initial search identified 73 studies and through manual searching of the reference lists a further three articles that matched the criteria were detected. After duplicate removal a total of 42 articles remained, which were narrowed by title, abstract and full-text review.

Six cohort studies were identified and included in the analysis with a total of 258 patients summing up to 1,167 naevi that were examined. An overview of the included studies in Tables 1, 2.

**Table 1:** Characteristics of the included studies.

**Study**	**Design**	**Population**	**No. of participants**	**No. of analysed patients**	**Age, median, y**	**History of melanoma**	**Family history of melanoma**	**Follow-up duration**
Akturk et al. (10) (Turkey)	Observational monocentric study	Women in the first trimester of pregnancy	77	56	26 ± 5.01	No	1 case	No follow-up
Rubegni et al. (11) (Italy)	Observational study	Women in the first trimester of pregnancy and non-pregnant women	70 (35 pregnant women and 35 control)	70	34	NR	NR	12 months postpartum
Zampino et al. (12) (Italy)	Observational monocentric study	Women in the first trimester of pregnancy	60	49	32.3± 3.5	NR	3 cases	6 months postpartum
Martins-Costa and Bakos (13) (Brazil)	Observational monocentric study	Pregnant women with a minimum of 10 naevi	18	18	33± 3.49	1	4 cases	No follow-up
Gunduz et al. (14) (Turkey)	Observational monocentric study	Women in the first trimester of pregnancy	21	21	26.8 ± 5.1	NR	NR	6 months postpartum in 3 cases
Strumia (15) (Italy)	Observational monocentric study	Primigravidas with naevi larger than 4 mm	12	12	36.2	No	No	No follow-up

*NR, not reported; no, number*.

**Table 2:** Clinical and dermatoscopic characteristics.

**Study**	**No. of analysed lesions (*n*)**	**Anatomical site (*n*)**	**Changes in lesion size, mean (mm)**	**Changes in colour**	**Dermatoscopic assessment**	**Pattern analysis**	**Dermatoscopic structures that changed during pregnancy**	**Developed melanoma (*n*)**	**Developed new naevi (*n*)**
Akturk et al. (10)	97	Front of body 27, face and neck 21, upper extremities 18, lower extremities 10, gluteus, and genital region 6	4.25 in first trimester vs. 4.59 in third trimester^*^	NR	Total dermoscopy score—TDS (ABCD rule) and pattern analysis	yes	Increased dots no. (49 in first trimester vs. 55 in third trimester^*^)	No	3/56
Rubegni et al. (11)	204	NR	NR	Darker globules, darker pigment network (increase in “CONTRAST” during pregnancy, decolouration postpartum)	DB-Mips® System	no	Changes in the colour of globules and network	No	NR
Zampino et al. (12)	86	Back	Non-significant change in the measures of the area between all three examinations	Lightening of the colour towards the end of pregnancy and postpartum	TDS (ABCD rule)	Yes	Less prominent pigment network, no. of vessels increased^*^	No	NR
Martins-Costa and Bakos (13)	703	Abdomen 101, back 178, anterior chest 146, lower limbs 114, neck 39, face 28, and upper limbs 93	55% of naevi enlarged	10.4% of cases increased pigmentation, 5,8% cases became hypopigmentation	Pattern analysis, FotoFinder Moleanalyzer system, total body photography^*^	Yes	Network changes 23.2%, new dots and globules 12.4%, new vascular structures 3.2%, new streaks 1.7%, and new structureless areas 1%	No	8/18
Gunduz et al. (14)	21	Back 10, face 6, neck 5	2 naevi increased in size	Colour changed in 2 lesions	TDS (ABCD rule)	No	Thickening of pigment network in 2 cases, development of radial streaming in 1 case, increase no., colour and size of dots and globules in 2 cases	No	NR
Strumia (15)	56	Abdomen 14, breast 5, forearm 18, back 6, hip and breast 9, and unknown 4	Increased size in the lesions on the breast and abdomen	No change	Pattern analysis	Yes	Lesions with pigment network became wide-meshed and clearer, lesions with globular patter developed brown globules at the periphery	No	Not included in the study

*NR, not reported; no, number*.

* *Statistical significant*.

### Study Design

All six included studies were observational cohort studies, the majority (five) being monocenter. None of the studies commented on the sampling technique and only one study (11) had a control group.

### Clinical Characteristics

The study population was made up of pregnant women, most in the first trimester of pregnancy. One study included only primigravidas (15), a further included only participants with a minimum of 10 naevi (13) and, as previously mentioned, only one study had an aged-matched control group (11). The median age of participants among the studies ranged between 26 and 36.2 years.

Personal history of melanoma was documented in three studies, only one participant having a positive history. Family history of melanoma was recorded in four studies and eight out of the 258 participants had a first-degree relative with a melanoma. The presence of dysplastic naevus syndrome was reported in one patient, while the presence of dysplastic naevus syndrome, congenital giant naevus and personal history of melanoma were exclusion criteria in one of the studies (10).

Three studies conducted a postpartum follow-up examination, the longest follow-up examination being at 12 months after delivery (11).

Fitzpatrick skin type was not documented in all studies, however, three studies were conducted in Italy, two in Turkey and one in Brazil, which included patients with skin type II, III, and IV.

Between the six studies a total of 258 participants were included, but only 226 participants with melanocytic lesion were examined by dermatoscopy.

The anatomical site of the lesions varied between the studies, with one study lacking to report the location of the lesions. The majority of the lesions were located on the front of the body, followed by the back, extremities, face, neck, and other regions such as genital, gluteal, hip (see Table 2).

In most studies, the participants were examined twice, in the first and third trimester, one study examining the participants in the second and third trimester. Three studies had a third follow-up examination, two at 6 months and one at 12 months postpartum 3 (11, 12, 14).

### Changes in Naevi During Pregnancy

An increase in size of the naevi was observed in most studies, however, only in one study the increase was statistically significant, with a mean increase from 4.25 mm in the first trimester to 4.59 mm in the third trimester (10) (Table 2). Enlarged naevi were observed in 55% of the participants in the study by Martins-Costa et al. (13), this being seen only in breast and abdomen naevi in the study by Strumia (15), naevi on the forearms and back remaining unchanged.

Development of new naevi was documented in two studies with incidences of 5.3 and 44% (13).

None of the 226 participants who had their naevi examined developed melanoma. In the study by Martins-Costa et al. (13), the authors excised one lesion during the follow-up period as this was a new fast growing lesion, which the study participant developed in the third trimester, the histopathologic examination reporting it as a dysplastic melanocytic naevus (10).

### Dermatoscopic Changes

Dermatoscopic assessment of lesions was done by total dermatoscopy score (TDS) using the ABCD rule in three studies (10, 12, 14), one of them using also pattern analysis, the remaining studies used either pattern analysis alone (15), pattern analysis, Fotofinder and Moleanalyzer system (13), and the DB-Mips® System (11). Pattern analysis was used as an assessment tool in four out of six studies. Changes in the dermatoscopic score used were noted in the three studies using the TDS, all three studies reporting a higher TDS for the lesions examined in the third trimester vs. the first. Two of the three studies reported that the TDS decreased or returned to base line at the 6 months postpartum follow-up examination.

Changes in colour were reported in four studies. Rubegni et al. (11) noted darker globules by dermatoscopy and increase in “contrast” during pregnancy, with decolouration of the same naevi at 12 months postpartum. Zampino et al. (12) reported of lightening of the colour towards the end of pregnancy and at 6 months postpartum. Martins-Costa et al. (13) observed increased pigmentation in 10.4% of the lesions and hypopigmentation in 5.8% during pregnancy.

With regard to specific patterns, one study reported structural irregularity with increased network irregularity and globules distribution as well as thickening of the pigment network.

Changes in the number of dots (increased number of dots) was the most frequent described dermatoscopic characteristic which changed during pregnancy. Akturk et al. (10) found a statistic significant difference between the number of dots in the first trimester in comparison to the third, dots were found in 49 naevi at the initial examination and in 55 naevi at the second examination in the third trimester. Martins-Costa et al. (13) found an increase of 12.4% of new dots and globules during pregnancy, network changes in 23.2% of their patients, new vascular structures in 3.2%, new streaks in 1.7% and new structureless areas in 1%. They also found that streak formation was more frequent with participants of skin type II compared to skin type III and IV, the difference being statistically significant. Increase in the number, colour and size of dots was also noted by Gunduz et al. (14) but only in two cases. They also found radial streaming in one case and thickening of the pigment network in further two cases. In the study by Strumia (15), lesions with an initial globular pattern developed peripheral brown globules a while lesions with an initial pigment network became wide-meshed and better defined. Development of new vessels was mentioned in two studies, Zampino et al. (12) noticing an increased number of dotted and comma vessels.

## Discussion

It has been reported that during pregnancy around 90% of women will undergo skin changes, including mole changes (16). The association between pregnancy and melanoma has been a hot topic for many years with reports supporting the idea that pregnancy increases the risk of melanoma (17). While single case reports of melanoma development during pregnancy (18) can be found in the literature, there are also several studies supporting the idea that pregnancy is not a risk factor for melanoma development (13, 19, 20).

In the present review, we included only cohort studies leaving out case reports.

Importantly, the main finding of our analysis is that none of the 258 women reported in the studies developed melanoma. However, this is not surprising: according to our calculations, the likelihood of this is at least 92%. Using a Bayesian model, we calculated that a study able to detect an increase in the incidence of melanoma should include between 30,000 and 100,000 women (Supplementary Figure 1).

The development of new lesions during pregnancy was mentioned in three out of the six studies with only two studies documenting objectively the incidence of new naevi developing; total body photography was used only in one of the studies for documenting new naevi. It is currently unclear whether these naevi developed physiologically given a relatively young age of the women or are indeed caused by hormonal changes during pregnancy. Based on our review, the former hypothesis seems to be the more convincing one.

Increase in size of the examined naevi was noticed in all but one study. While some studies found the majority of the lesion increased in size (13), another study found this only in lesion on anatomical sites who undergo distension during pregnancy such as abdomen or breast (15). In the study by Gunduz et al. (14), which included lesion on the back, face, and neck, increase in size was observed only in 2 naevi. It stands to reasons, that in naevi which increase in size during pregnancy and that are located in anatomic sites prone to distension, such as abdomen and breasts, the main cause for this change is mechanical rather than hormonal.

Changes in colour were addressed in four out of the six studies. An interesting finding was the observation that some lesions become more hyperpigmentated during the pregnancy, but the colour of the lesions lighten towards the end of the pregnancy or postpartum. It has been shown that melanocytes as well as melanoma cells express functional estrogen and androgen receptors and that their activity is influenced by several factors including anatomic location (21). In pregnancy the number of estrogen and progesterone receptors on melanocytes increases and is responsible for the pigmentary changes that can be observed during pregnancy (22).

Three studies used the TDS for assessing the naevi dermatoscopically and in all three studies the TDS was higher in the third trimester compared to the first one. When following up the patients postpartum, two studies, Zampino et al. (12) and Gunduz et al. (14), mentioned a decrease of the TDS and return to the baseline TDS (11).

The most frequent observed dermatoscopic change among the studies was the increase in the number of dots. In Strumia's study, it was noticed that naevi with a globular patter developed more peripheral brown globules and the studies by Rubegni et al. (11) and Gunduz et al. (14) also mention changes and increased number of dots and globules (15). The presence of peripheral dots and globules seen with dermatoscopy is generally a sign of naevus growth (23) at young age, however, these pregnancy-related observations could be also result of the mechanical distension of the skin resulting in an upward movement of the basal layer with junctional or dermal nests becoming more easily visible by dermatoscopy (15).

Network changes have been observed in five out of the six studies. While some studies reported a less prominent network or a change into a broader wide-meshed network, others found that the network became more thickened and in some cases even developed radial streams, a sign generally associated with a malignant phenotype. Again, most of these changes may be attributable to the distension of some body parts, resulting in a thinned epidermis and subsequently, better visualisation upon dermatoscopy.

Vessel formation during pregnancy was reported in two studies, in one being observed in 12.4% of the cases (13), but appears to decrease again after delivery in the post-partum period. It is known that physiologic vascular changes happen during pregnancy and that these are a response to the increased production of estrogens and human chorionic Gonadotrophin, which are placental hormones (24, 25). The dermatoscopic vascular changes noticed during pregnancy may therefore become visible due to the vascular modifications such as vasodilation and proliferation of dermal blood vessels.

Limitations of the present study are the relatively low number of women included and the lack of appropriate control groups, which would have facilitated a better statistical analysis of physiological and truly pregnancy-related changes. Additionally, the included studies are heterogeneous, thus not allowing head-to-head comparisons between them. Only one study used a control group and no study displays raw data facilitating a reanalysis, comparison or merging of information.

Robust studies of dermatoscopic evaluation of naevi in pregnant women are needed to determine high-risk dermatoscopic characteristics, which would improve the diagnostic accuracy of benign vs. malignant melanocytic lesion in this population.

## Author Contributions

IC and IZ contributed to the study conception. IC, MT-H, LU, and IZ contributed to the study design. Data collection and analysis was performed by IC, LU, and MT-H. IC, MT-H, LU, and IZ prepared and reviewed the manuscript. All authors contributed to the article and approved the submitted version.

## Conflict of Interest

The authors declare that the research was conducted in the absence of any commercial or financial relationships that could be construed as a potential conflict of interest.

## Publisher's Note

All claims expressed in this article are solely those of the authors and do not necessarily represent those of their affiliated organizations, or those of the publisher, the editors and the reviewers. Any product that may be evaluated in this article, or claim that may be made by its manufacturer, is not guaranteed or endorsed by the publisher.
