# Supplementary material for: Dermatoscopic Features of Naevi During Pregnancy—A Mini Review
Source: Front Med (Lausanne). 2021 Aug 9;8:727319. doi: 10.3389/fmed.2021.727319 (PMC8381148; doi:10.3389/fmed.2021.727319)
Supplement: Supplementary file 1 [file Data_Sheet_1.docx]

Supplementary Material

**Supplemental Table 1.** Quality assessment of included studies according to the Newcastle-Ottawa Scale

| Study |  | Selection | Comparability | Outcome |
| --- | --- | --- | --- | --- |
| Zampino et al, 2006 |  | ★★ |  | ★ |
| Aktürk et al, 206 |  | ★★ |  | ★ |
| Rubegni et al, 2006 |  | ★★★ | ★ | ★★★ |
| Martins-Costa G.M, Bakos R, 2019 | | ★★ |  | ★ |
| Gunduz et al, 2003 |  | ★★ |  | ★ |
| Strumia, 2002 |  | ★★ |  | ★ |

**Supplemental Table 2.** Risk of bias assessment of included studies according to the Cochrane Risk of Bias Tool

| Study | Random sequence generation | Allocation concealment | Blinding participants and personnel | Blinding outcome assessment | Incomplete outcome data | Selective reporting |
| --- | --- | --- | --- | --- | --- | --- |
| Zampino et al, 2006 | no | no | no | no | no | no |
| Aktürk et al, 206 | no | no | no | no | no | no |
| Rubegni et al, 2006 | no | no | no | no | no | no |
| Martins-Costa G.M, Bakos R, 2019 | no | no | no | no | no | no |
| Gunduz et al, 2003 | no | no | no | no | no | yes |
| Strumia, 2002 | no | no | no | no | no | no |

**Discussion**

As mentioned in our manuscript, none of the 258 women reported in the studies developed melanoma. Considering an age-standardised incidence for melanoma between 9 and 33 per 100,000 people in the female population (1), even when considering the highest incidence of melanoma, the likelihood of no woman developing a melanoma in our analysis lies at 92% (calculated using the binominal distribution, where (1-33/100000)^258^=0.918). In order to accept or reject a hypothetical increase in the incidence of melanoma of 17% (considered based on the increase in mortality from melanoma in pregnant women), a study would have to include between 30,000 and 100,000 women, taking a Bayes factor of at least 3 which is the minimal value for the evidence to be considered substantial using the Jeffrey’s scale (2, 3). See Supplemental Figures 1A, 1B and 1C.

**Supplemental Figure 1A.** Evidence (as Bayer factor) vs sample of size needed to prove or disprove an incidence 17% higher, in case 0, 1, or 5 cases of melanoma are eventually diagnosed. An average incidence of 21 per 100,000 people and year in the female population, with error envelopes for incidences between 9 and 33 per 100,000 people and year. The red dashed line shows the evidence for a sample of 226 women


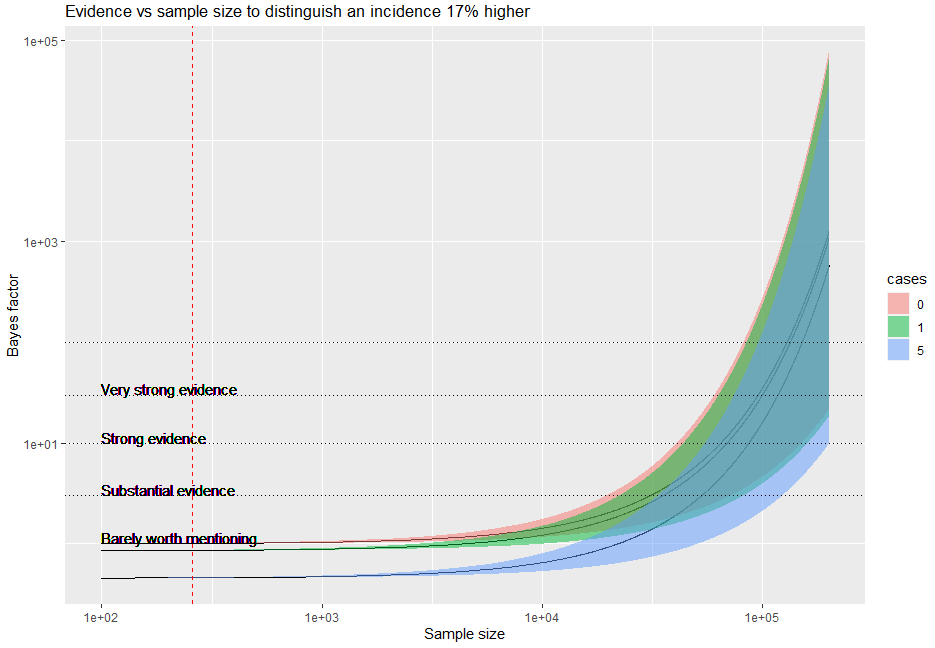


**Supplemental Figure 1B.** Same information, considering as alternative hypothesis with an incidence 50% higher.


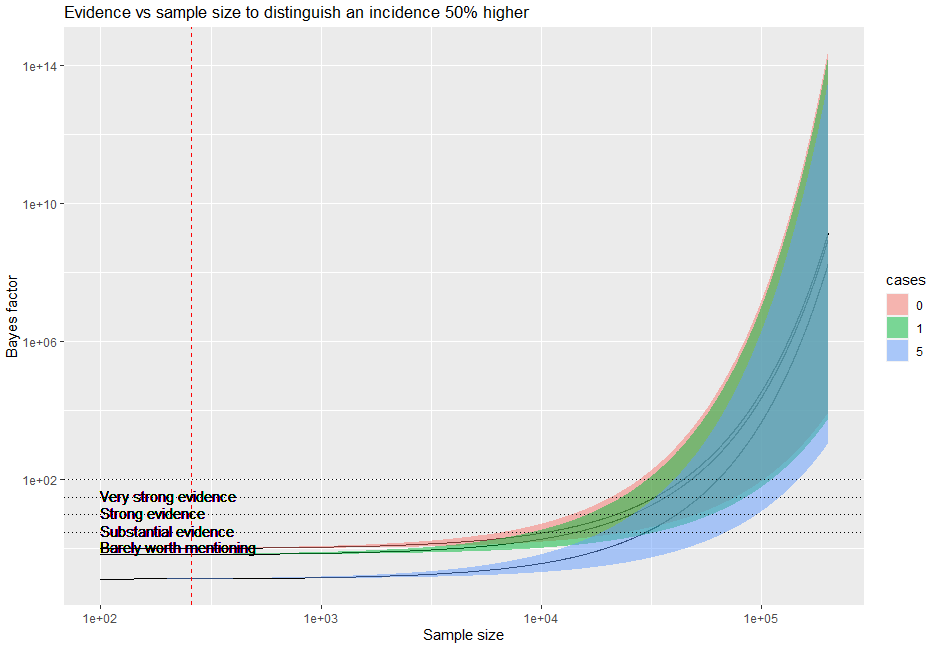


**Supplemental Figure 1C.** Same information, considering as alternative hypothesis with an incidence 5% higher.


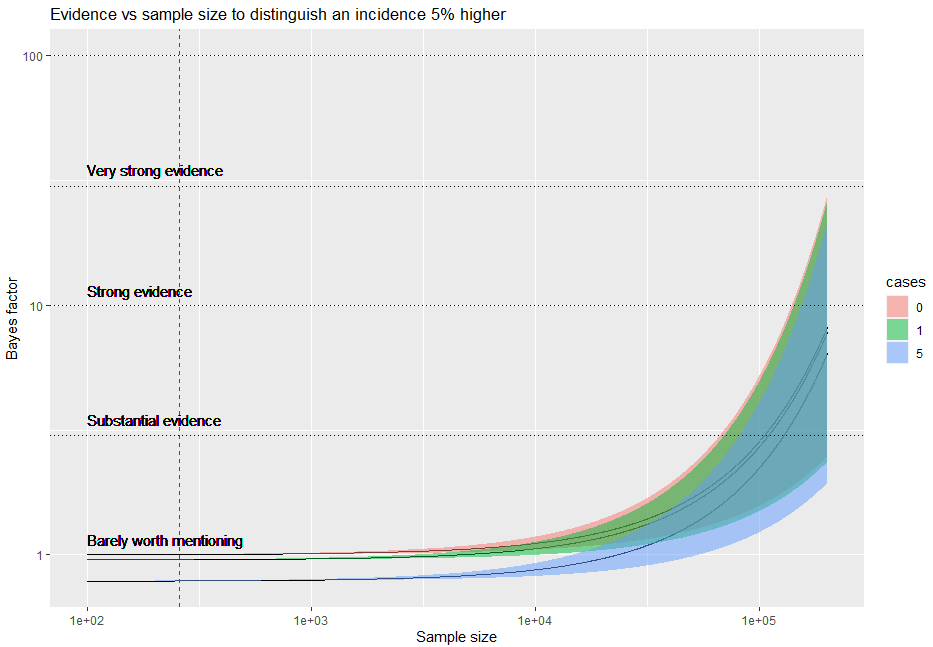


**References**

1. Fund WCR. Data: Skin cancer statistics 2021 [Available from: <http://www.wcrf.org/dietandcancer/cancer-trends/skin-cancer-statistics>. accessed on 24th May, 2021.

2. Z. D. How Bayes factors change scientific practice. Journal of Mathematical Psychology. 2016;72:78-89.

3. Ly A VJ, Wagenmakers EJ, . Harold Jeffrey's default Bayes factor hypothesis tests: Explanation, extension, and application in psychology. Journal of Mathematical Psychology. 2016;72:19-32.
